# Bibliometric study of research trends in dysphagia complicating following anterior cervical spine surgery

**DOI:** 10.3389/fsurg.2025.1550816

**Published:** 2025-04-03

**Authors:** Shang Qisong, Xiang Wei, Wu Yuanyuan, Song Xinghua

**Affiliations:** ^1^ The Third Department of Spine Surgery, The Sixth Affiliated Hospital of Xinjiang Medical University, Orthopaedic Hospital of Xinjiang Uygur Autonomous Region, Urumqi, China; ^2^ Department of Spine Surgery, The Third Affiliated Hospital of Shihezi University, Shihezi, Xinjiang, China

**Keywords:** bibliometrics, anterior cervical spine surgery, VOSviewers, CiteSpace, dysphagia

## Abstract

**Background:**

The aim of this study was to assess the global research status and trends in the occurrence of dysphagia after cervical spine surgery using bibliometrics.

**Methods:**

All relevant research publications on dysphagia occurring after cervical spine surgery were retrieved from the Web of Science Core Collection database. Literature coupling, co-citation and co-occurrence analyses were subsequently visualised using VOSviewer, CiteSpace. WPS Office was applied for data summary processing.

**Results:**

Between 2000 and 2023, a total of 477 clinical studies met the inclusion criteria. The number of global publications has steadily increased in four stages over the last 19 years, with the United States having the most publications (=194), followed by China (=134) and South Korea (=34). The most contributing institutions were UNIVERSITY OF CALIFORNIA SYSTEM in the USA (*n* = 24) and SICHUAN UNIVERSITY in China (n-21). The most distinguished scholar was Liu,Hao (*n* = 15), followed by Albert (*n* = 10) and Yang,Yi (*n* = 9). Ten of the most cited papers were cited more than 65 times. The most important journal for research on the occurrence of dysphagia after cervical spine surgery was SPNIE (*n* = 445), followed by EUR SPINE J (*n* = 337) and SPINE J (*n* = 322), which analysed a number of factors including anatomy, patient information and the use of inbuilt objects. The top 20 most commonly used keywords were identified from 750 author keywords, with the highest number being dysphagia (*n* = 303), followed by fusion (*n* = 183) and spine surgery (182). In parallel with time zone and cluster analysis we found multiple high frequency keywords that appeared as early as 2006 and have continued to the present day, reflecting the enthusiasm of a large number of scholars who have researched this topic.

**Conclusion:**

This bibliometric study analyses the global research hotspots and trends in postoperative cervical spine complication dysphagia in terms of study type, patient information, surgical modality, surgical segment, most popular keywords, most cited papers, journals, authors, institutions, and countries, to guide future practice and direction, in order to help understand how to effectively prevent or reduce the incidence of this postoperative complication so as to achieve the goal of lowering the patient's healthcare costs, to balance social medical resources and reduce the financial burden of the government.

## 1 Introduction

Anterior cervical spine surgery is widely used in clinical practice for the treatment of cervical spine degeneration and traumatic diseases due to its safety, high effectiveness and low complications (1). However, with the increase in the number of surgical cases, the reports of surgery-related complications are also increasing year by year (2), and dysphagia is one of the common complications of anterior cervical spine surgery, which has been reported in the literature to have occurred in 1%–79% (3), referring to a subjective discomfort that occurs when the normal transport of food from the mouth to the cardia is obstructed. It has been reported in the literature (4) that the majority of patients undergoing anterior cervical spine surgery have no or only mild dysphagia, and that the symptoms of dysphagia gradually diminish or disappear 6–12 months after surgery. However, persistent or severe dysphagia can create a series of negative problems; however, some patients have difficulty tolerating this dysphagia and often require outpatient or inpatient treatment, which increases the patient's healthcare costs (5); Several previous studies (6–10) found significant associations between age, female gender, smoking, body mass index, number of surgical segments, surgical segment location, diabetes, operation time, and intraoperative use of plate insert with dysphagia. In recent years, a large number of scholars have conducted research on the influencing factors and etiology of dysphagia after anterior cervical spine surgery, in order to provide early intervention and effectively avoid guiding suggestions.

Bibliometrics research is a method of visualising and analysing a large amount of relevant literature on an international scale for a certain topic, which can be used to qualitatively and quantitatively analyse the scientific research results in a specific field within a certain time period (11), we can use the bibliometric tools VOS viewer 1.6.20, CiteSpace 6.2.R6 for the countries, institutions, journals, authors, keywords, reference details for analysis and visualisation such as co-occurrence, emergence, etc., and can also draw double overlay diagrams to give readers a more intuitive understanding of the state of research in the field, and these tools have been widely used in scientific research.He et al. (12) who conducted a bibliometric analysis of trends in global post-stroke dysphagia rehabilitation studies, and Li et al. (13) who conducted a global post-stroke pneumonia study Trend bibliometric analysis, but no bibliometric analysis of dysphagia after anterior cervical spine surgery, research on the influencing factors of dysphagia after anterior cervical spine surgery and the related preventive measures can effectively reduce and avoid the occurrence of such complications, in order to fill this knowledge gap, the present study aims to visualise the scientific outputs of the research on dysphagia after anterior cervical spine surgery in the past nineteen years (from 2005–2023) A visual bibliometric analysis was carried out to identify the main contributors and the current state of research, as well as to look at research trends and future preventive and curative measures in the field.

## 2 Subjects and methods

### 2.1 Data sources and the retrieval strategy

**Figure 1:**
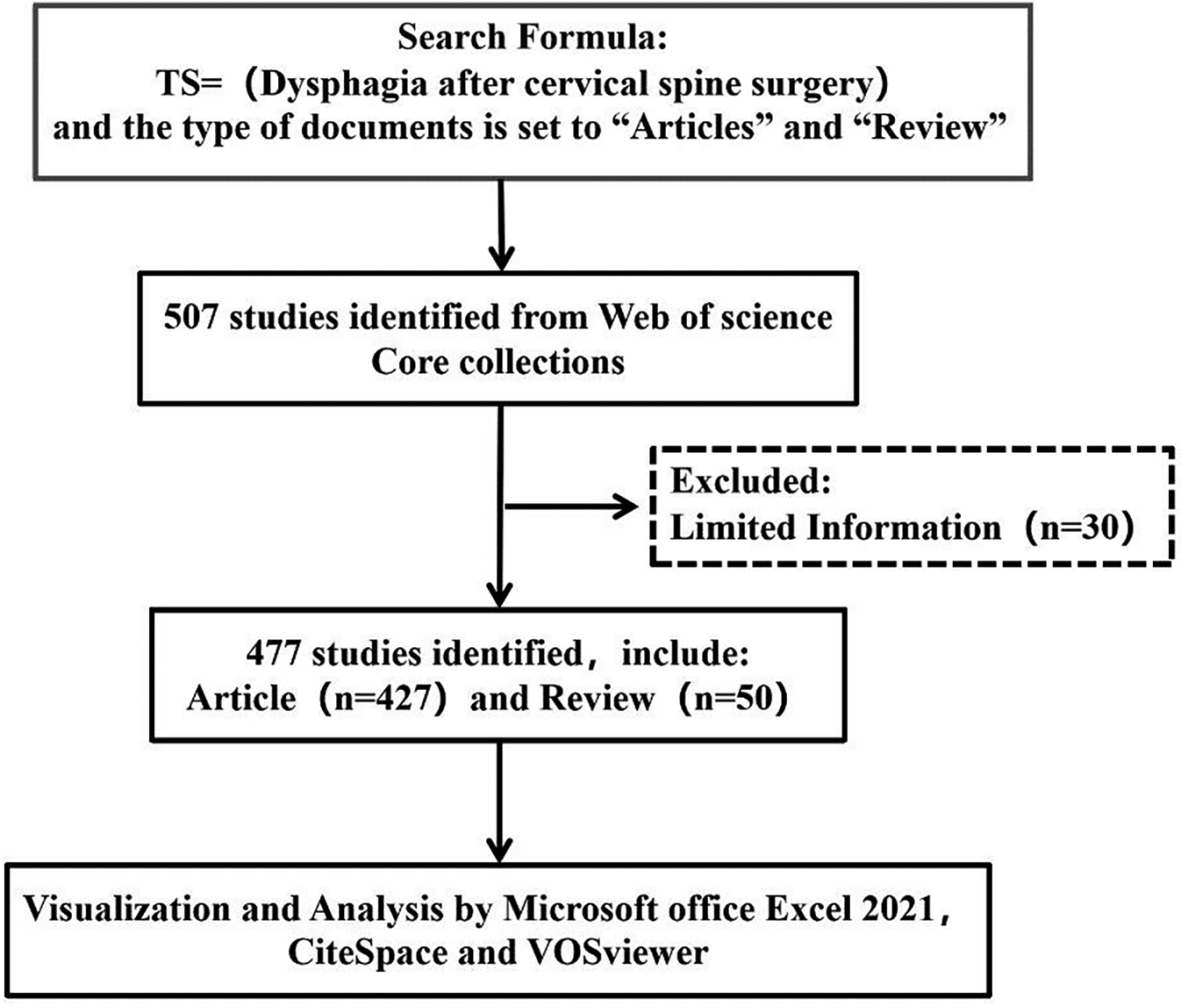
Flow-chart of the retrieval strategy.

In order to conduct a more comprehensive literature search for this study and to avoid literature search bias, the first author and co-first author on 2024-11-01 firstly used PubMed to determine the subject terms and their synonyms for the search, and then searched the literature based on the Web of Science Core Collection (WOSCC) with dysphagia after anterior cervical spine surgery. (https://www.webofscience.com/wos/woscc) and dysphagia after anterior cervical spine surgery, the search strategy was TS = (Dysphagia after cervical spine surgery), the type of literature was Articles and Review, and the language was English. The flowchart of the search strategy is shown in Figure 1. Two authors cross-searched for duplicate articles, letters, retractions, errata, conference proceedings and abstracts.

### 2.2 Research method

#### 2.2.1 Software selection

The data analysis in this paper was performed using Excel, CiteSpace 6.1.R6 (Advanced Edition) and VOSviewer 1.6.20. CiteSpace Is an information visualization software developed by Chen's team using Java language. It combines the dual properties and characteristics of “chart” (visual chart) and “spectrum” (citation analysis map). Its biggest advantage is that, by drawing a series of visual maps, it forms an in-depth analysis of the potential dynamic mechanism of discipline evolution, and further reveals the research structure and development trend of a certain discipline field. Leiden University, based on similarity visualisation technology, VOSviewer is a software tool for building and visualizing maps of scientific knowledge. It is mainly used for the analysis of bibliometric data. By mining the relationship among various elements in the literature (such as keywords, authors, journals, institutions, etc.), it can present the structure, development trend and hot spots of the research field in an intuitive and visual way (14).

#### 2.2.2 Inclusion and exclusion criteria

##### 2.2.2.1 Inclusion criteria

(1) Participants: Patients undergoing anterior cervical spine surgery; (2) Intervention: Anterior cervical spine surgery serves as a primary treatment. (3) Control: Controls between the different surgical methods; (4) Outcome: Dysphagia after cervical spine surgery; (5) Study design: Articles and Review.

##### 2.2.2.2 Exclusion criteria

(1) case report and conference summary; (2) literature with incomplete original data; (3) literature published repeatedly.

#### 2.2.3 Data import

After completing the steps of literature search and screening, we need to convert and export the valid literature after eliminating irrelevant literature. Firstly, these selected documents are exported in plain text format (this format usually maintains the original content of the documents, which is easy to read and edit) and tab-delimited format (this format distinguishes different fields by specific characters such as comma, tab, etc., which is convenient for data import and processing). In the export process, special attention is paid to the need to choose the comprehensiveness of the record content, that is, not only to include the full record information of the literature itself (this covers key elements such as the title of the literature, authors, abstracts, keywords, publication information, and so on), but also to include a list of references cited by the literature, this is because the references are an important basis for understanding the background of the literature, and tracing the research lineage. Access to the JAVA programming environment, this is because both the CiteSpace 6.1.R6 (advanced) software and the VOSviewer 1.6.20 software support data import via programming interfaces or specific file formats.

#### 2.2.4 Parameter setting

The parameters of CiteSpace are set as follows: (1) the time span is from January 2005–October 2024, annual section =1; (2) term source = title/abstract/author keywords/keywords; (3) node type = country/organization/author/keywords/reference; (4) threshold selection criteria = the first 50 items of each time slice. VOSviewer 1.6.20 According to the literature database, “Choose type of data” is drawn based on the literature data, and the parameters are read from the literature database files.

### 2.3 Research indicators

Excel software was used to chart the trend of the number of publications in order to statistically analyse the output of the literature on dysphagia complicated by anterior cervical spine surgery. Meanwhile, with the help of CiteSpace 6.1.R6 (advanced) and VOSviewer 1.6.20, we conducted in-depth co-occurrence and cluster analyses for the screened literature in terms of multiple dimensions, such as country/region, research organisation, author group, journal as well as co-cited journals, and keywords. Through these analyses, we successfully generated various types of maps, which visually reveal the current research status of the field and the research hotspots at different historical stages. In addition, the results of these analyses provide us with strong data support for predicting future research trends in this field.

## 3 Result

### 3.1 Analysis of the number of publications and citations

**Figure 2:**
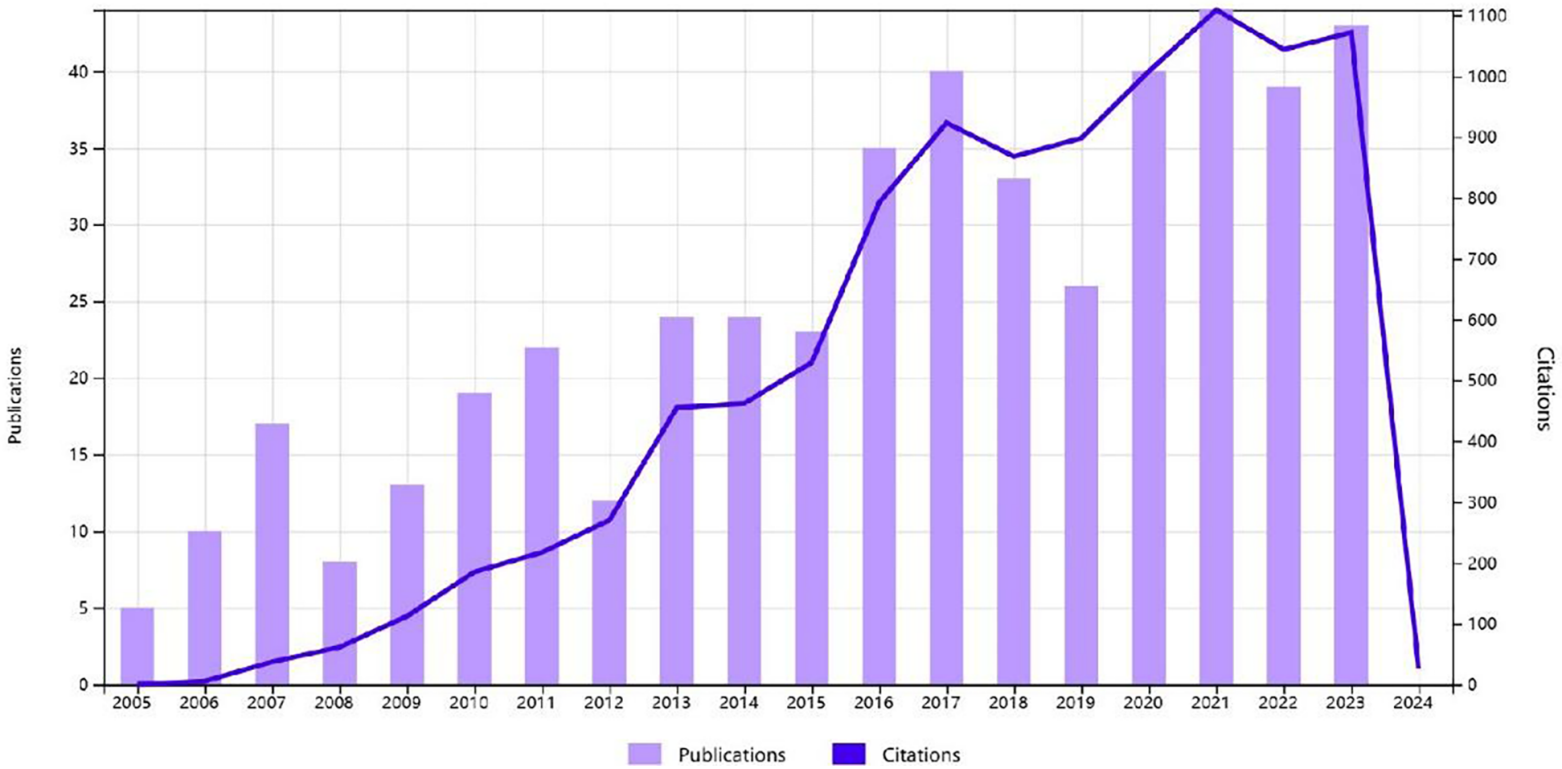
The number of publications in the literature on postoperative cervical spine complicating dysphagia in the last 20 years in the Web of science core collection database.

According to our search formula, a total of 477 studies on postoperative dysphagia after cervical spine surgery were published in the past nineteen years, including 427 Articles and 50 Reviews. The number of publications and citations has increased over the years, and the number of publications in each period reflects the current state of development of the field (Figure 2). The number of publications in the first and second phases can be roughly divided into four phases, the first phase 2005–2007, the second phase 2008–2012, the third phase 2013–2015, and the fourth phase 2016–2023. The number of publications in the first and second phases showed two waves of small peaks, reflecting that relevant scholars began to notice the phenomenon of concomitant dysphagia after cervical spine surgery and started to review, analyse, and cohort the causes of this phenomenon. The number of articles published in the third stage shows a steady growth trend, but the number of cited literature in this stage increases year by year, reflecting that more and more scholars have a consensus in this area and are conducting research. The number of articles published and the number of citations in the fourth stage are at a high level, reflecting that a large number of scholars have begun to conduct in-depth excavation of the factors affecting the concurrent dysphagia after cervical spine surgery.

### 3.2 National and institutional analysis

**Figure 3:**
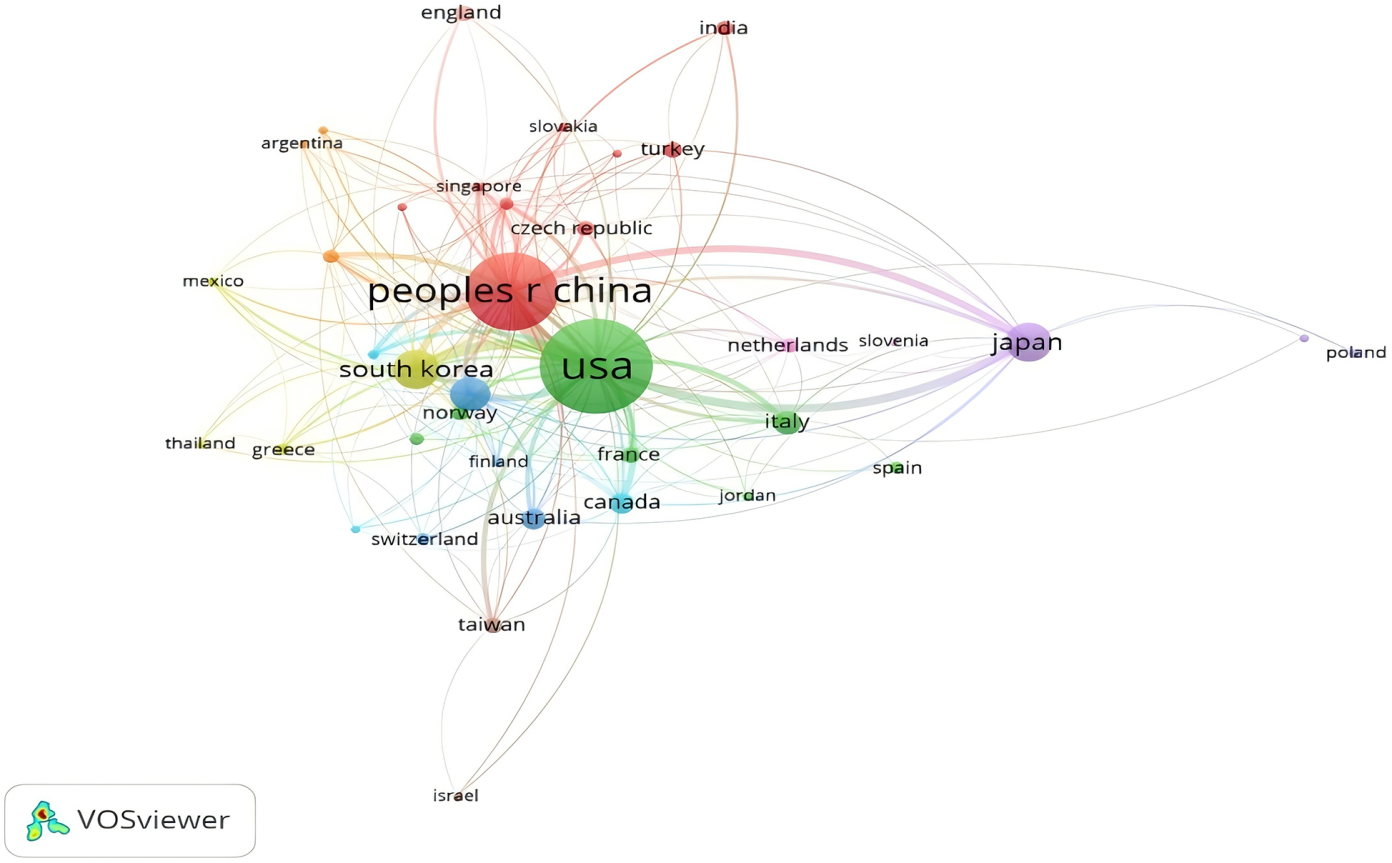
National network map of postoperative cervical spine complicating dysphagia literature postings in the Web of science core collection database.

**Figure 4:**
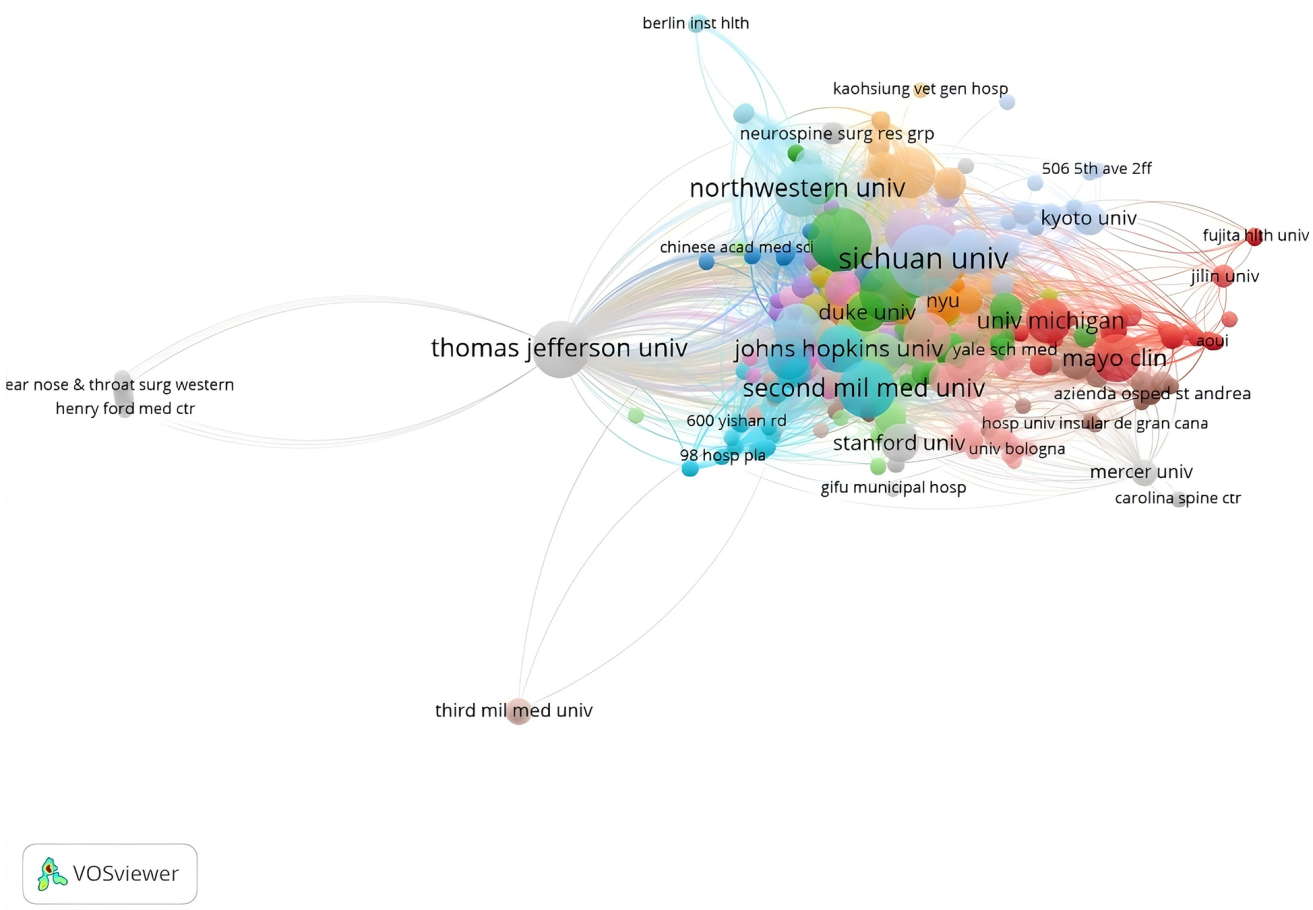
Network network of cervical spine postoperative dysphagia in Web of science core collection database.

The literature came from various institutions around the world and the top ten countries and institutions are shown in the table (Table 1). The country with the highest number of publications is the United States (*n* = 194, or 40.67%), followed by China (*n* = 134, or 28.10%), Korea (*n* = 34, or 7.13%), Japan (*n* = 33, or 6.92%), and Germany (*n* = 25, or 5.24%). The United States and China had the highest number of publications, both with more than 100 articles. The research institutions with the highest number of publications were UNIVERSITY OF CALIFORNIA SYSTEM with 24 (5.031%), followed by SICHUAN UNIVERSITY with 21 (4.403%), JEFFERSON UNIVERSITY with 20 (4.193%), NAVAL MEDICAL UNIVERSITY, 20 articles (4.193%); and RUSH UNIVERSITY, 18 articles (3.774%). Institutions within the top ten ranked institutions accounted for 21%. The countries (Figure 3) and institutions were visualised according to the number of publications greater than 1 (Figure 4), with close collaboration between different countries and institutions.

**Table 1:** Top 10 countries with the highest number of publications on post-cervical spine surgery complicating dysphagia in the Web of science core collection database.

Rank	Country	Counts	Affiliations	Counts
1	USA	194 (40.67%)	University of California System	24 (5.031%)
2	Peoples R China	134 (28.10%)	Sichuan University	21 (4.403%)
3	South Korea	34 (7.13%)	Jefferson University	20 (4.193%)
4	Japan	33 (6.92%)	Naval Medical University	20 (4.193%)
5	Germany	25 (5.24%)	Rush University	18 (3.774%)
6	Italy	12 (2.52%)	Rothman Institute	14 (2.935%)
7	Australia	10 (2.09%)	Johns Hopkins University	13 (2.725%)
8	Canada	10 (2.09%)	Hosp Special Surg	12 (2.516%)
9	England	6 (1.26%)	Northwestern University	11 (2.306%)
10	France	6 (1.26%)	Cleveland Clinic Foundation	10(2.096%)

### 3.3 Co-occurrence analysis of journals and cited journals

**Figure 5:**
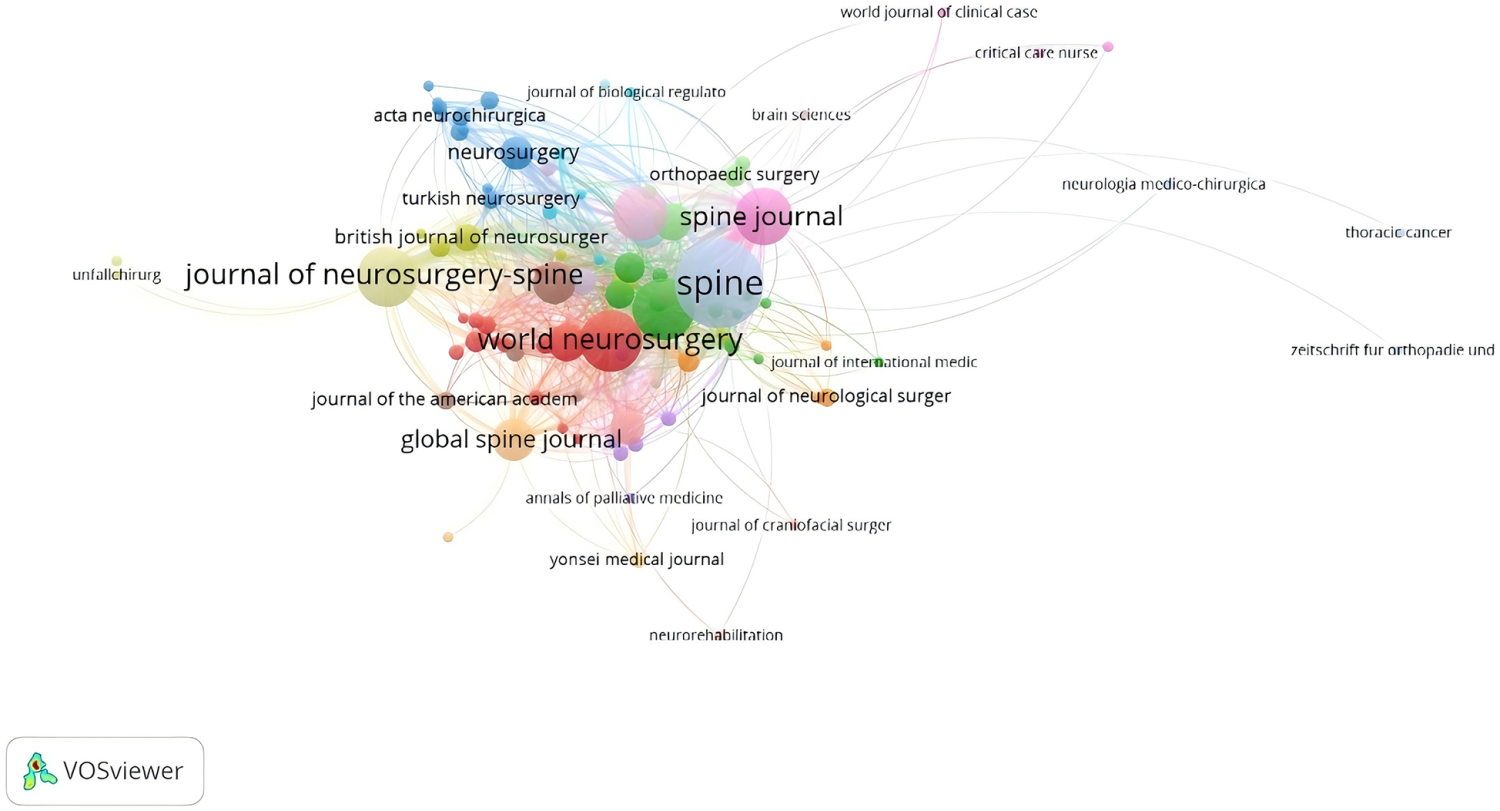
Journal network map of postoperative cervical spine complicating dysphagia literature postings in the Web of science core collection database.

**Figure 6:**
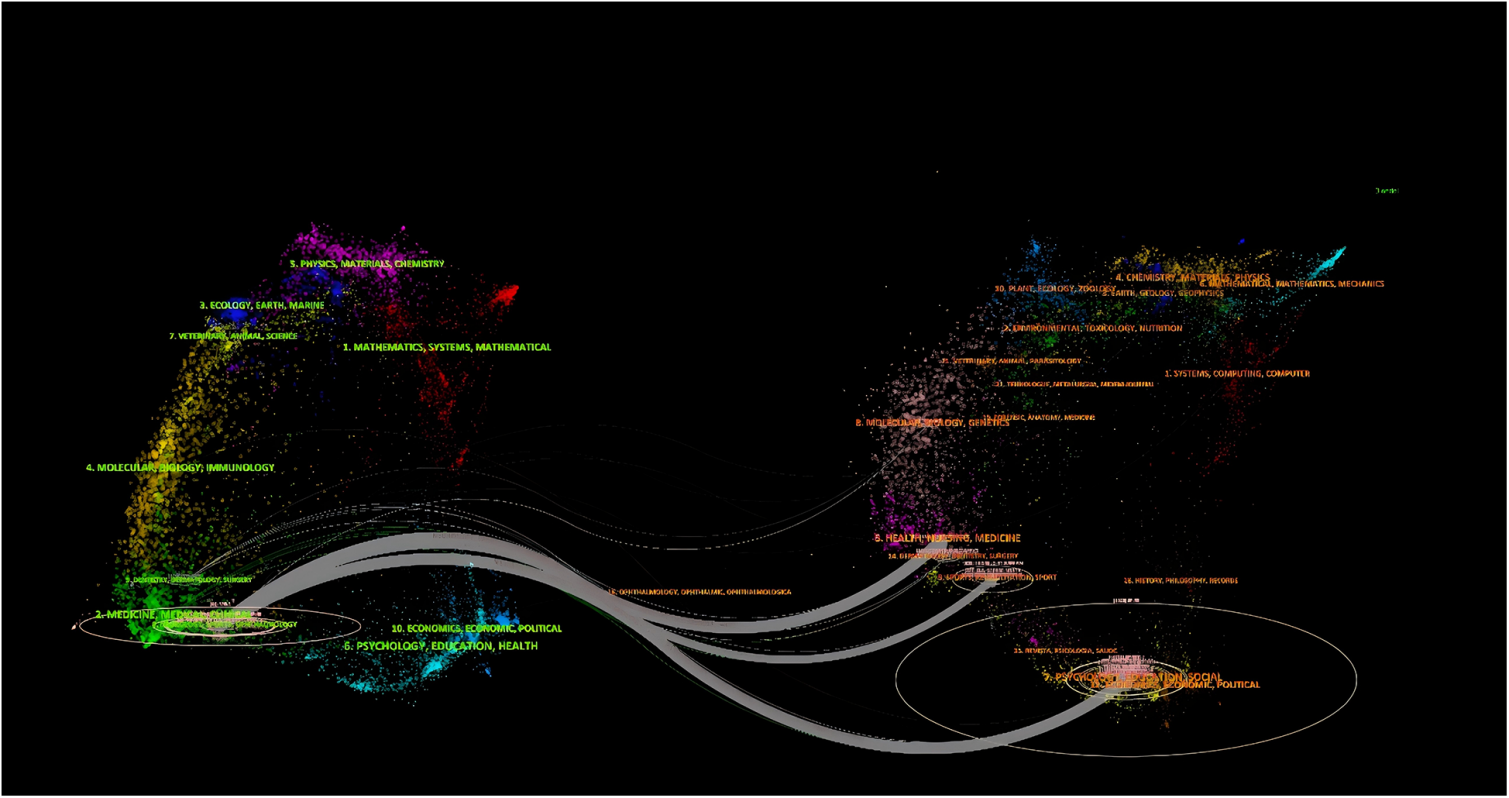
Network diagram of cross-citation relationships between journals and cited journals for post-cervical spine surgery complicating dysphagia in the Web of science core collection database.

Based on the network co-occurrence graph of journals in the published literature it can be seen that SPINE is not only the journal with the highest number of publications (Table 2), but also the most cited journal (*n* = 445), followed by EUR SPINE J (*n* = 337), SPINE J (*n* = 322), and only these three journals were cited more than 300 times (Table 2). The journal with the highest impact factor was J BONE JOINT SURG AM (IF = 5.2999), followed by NEUROSURGERY (IF = 4.8003), and SPINE J (IF = 4.5003).SPINE was also the journal with which the strongest intercitation relationship existed. A double graphical overlay of the journals shows the cross-citation relationships between the journals and the cited journals (Figures 5, 6), with the citing journals on the left and the cited journals on the right. As shown, the grey path is the primary citation path, representing research published in clinical medicine/sports hospitals/neurology/ophthalmology journals primarily cited by literature in health care nursing/dermatology/oral medicine/sports rehabilitation medicine/socio-educational psychology and economics and political science journals.

**Table 2:** Network diagram of the top 10 journals in the Web of science core collection database for postoperative cervical spine complicating dysphagia.

Rank	Cocited Journal	Counts	IF	Cocited Reference	Citations	IF
1	Spine	445	3.468	Bazazr, 2002, Spine, v27, p2453, doi 10.1097/00007632-200211150-00007	219	3.468
2	Eur Spine J	337	2.8002	Smith-Hammond CA, 2004, Spine v29, p1441, doi 10.1097/01.brs.0000129100.59913.ea	121	3.468
3	Spine J	322	4.5003	Fountas KN, 2007, Spine, v32, p2310, doi 10.1097/brs.0b013e318154c57e	116	3.468
4	J Spinal Disord Tech	299	1.726	Lee MJ, 2007, Spine J, v7, p141, doi 10.1016/j.spinee.2006.02.024	102	4.5003
5	J Bonejoint Surg AM	299	5.2999	Riley IH, 2005, Spine v30, p2564, doi 10.1097/01.brs.0000186317.86379.02	98	3.468
6	J Neurosurg-Spine	292	2.8002	Frempong-boadu A, 2002, J Spinal Disord Tech,vl5, p362, do110.109700024720-200210000-00004	93	1.726
7	J Neurosurg	219	4.0998	Lee MJ, 2005, J Spinaldisord Tech, vl8, p406, doi 10.1097/01.bsd.0000177211.44960.71	92	1.726
8	Neurosurgery	208	4.8003	Rihn JA, 2011, Clin Orthop Relatr, v469, p658, doi 10.1007/s11999-010-1731-8	81	4.1998
9	Clinorthop Relatr	173	4.1998	Smith GW, 1958, J Bone Joint Surgam v40, p607, doi 10.2106/00004623-195840030-00009	71	5.2999
10	Worldneurosurg	169	1.9999	Riley IH, 2010, Spine, v35, ps76, doi 10.1097/brs.0b013e3181d81a96	67	3.468

### 3.4 Reference co-occurrence analysis

**Figure 7:**
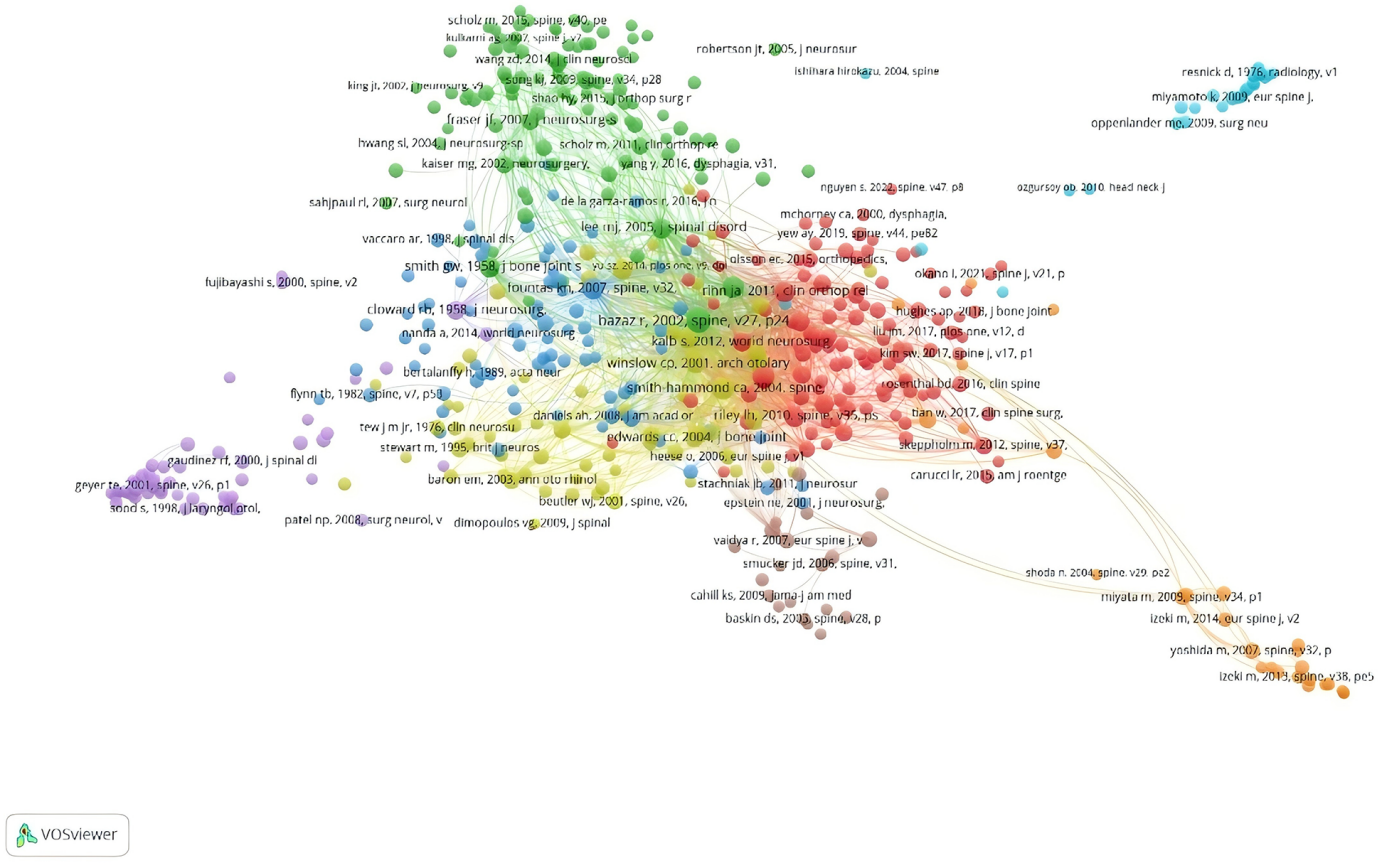
Network co-occurrence map of references with post-cervical spine surgery complicating dysphagia in the Web of science core collection database.

**Figure 8:**
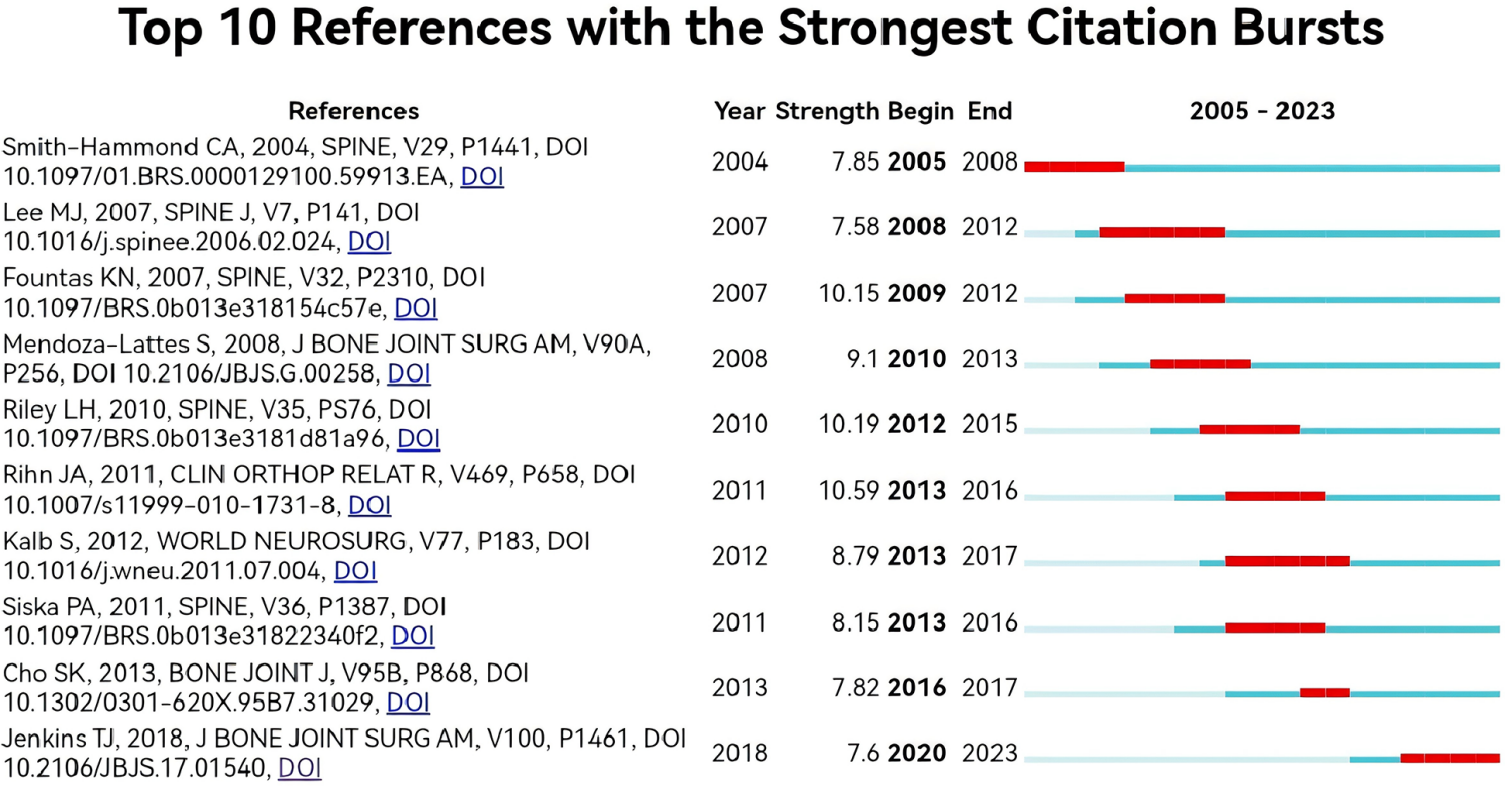
Network diagram of the top 10 literature emergence analyses of post-cervical spine surgery complicating dysphagia in the Web of science core collection database.

The most cited paper was Bazaz R, 2002, SPINE, v27, p2453, doi 10.1097/00007632-200211150-00007 (*n* = 219, IF = 3.468) (Figure 7), followed by Smith-Hammond ca, 2004, SPINE v29, p1441, doi 10.1097/01.brs.0000129100.59913.ea (*n* = 121, IF = 3.468), an article published in SPINE J (Lee Mj, 2007, SPINE J, v7, p141, doi 10.1016/j.spinee.2006. 02.024) has also been cited more than 100 times, and the reference network co-occurrence graph shows that the citations are relatively balanced across references, with no relatively large nodes (Figure 8). We used Citespace to analyse the emergence of references, and listed the 10 literature with the highest outbreak strength, of which the top one was published by Rihn JA in CLIN ORTHOP RELAT R in 2011 (Strength = 10.59), reflecting the high heat of this literature during the four years from 2013–2016. Overall, these 10 literature outbreaks ranged in strength from 7.58–10.59, with a persistence time of 3–7 years.

### 3.5 Analysis of co-occurrence and emergence of authors and cited authors

**Figure 9:**
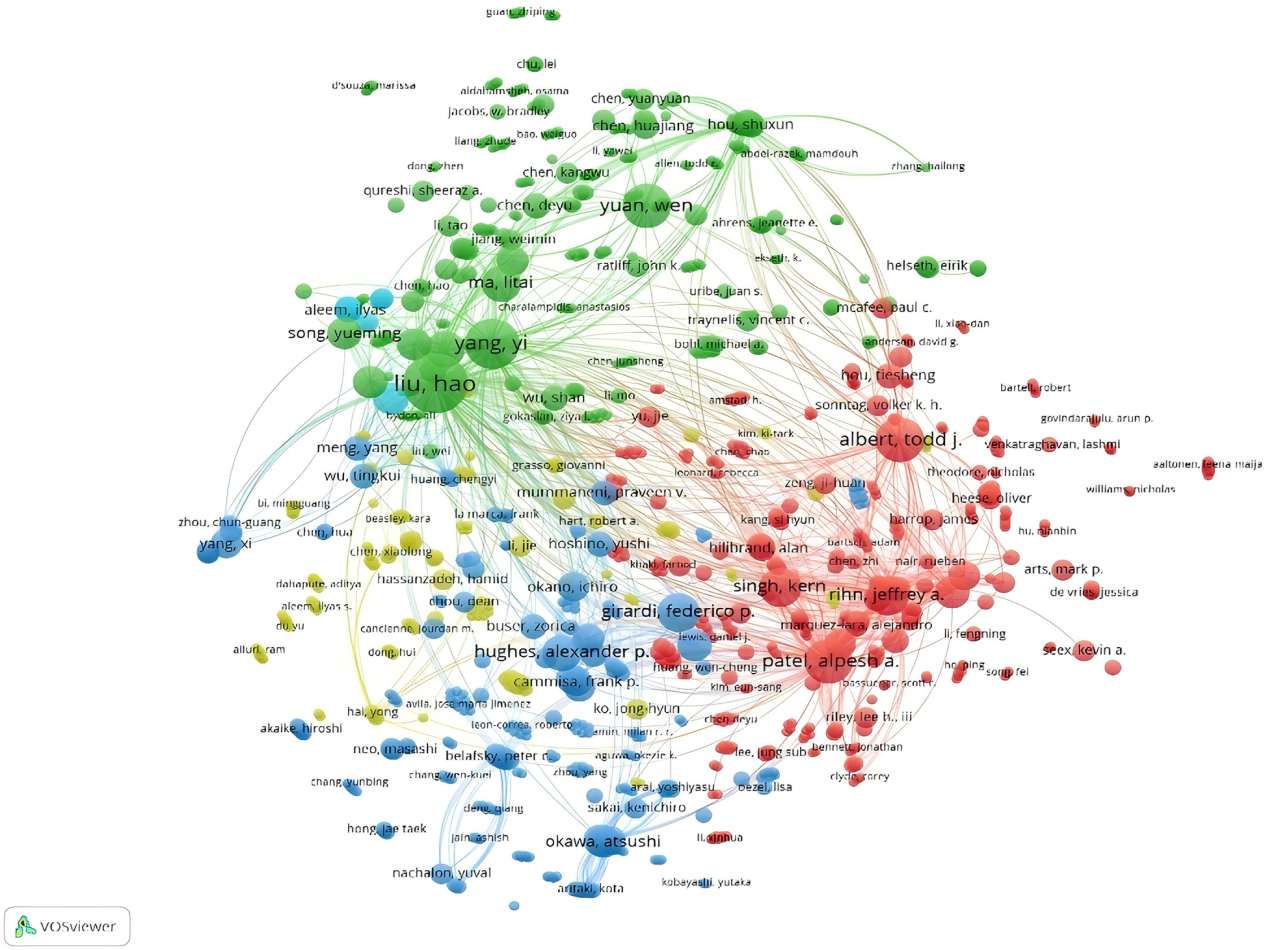
Network diagram of collaboration between authors of post-cervical spine surgery complicating dysphagia in the Web of science core collection database.

**Figure 10:**
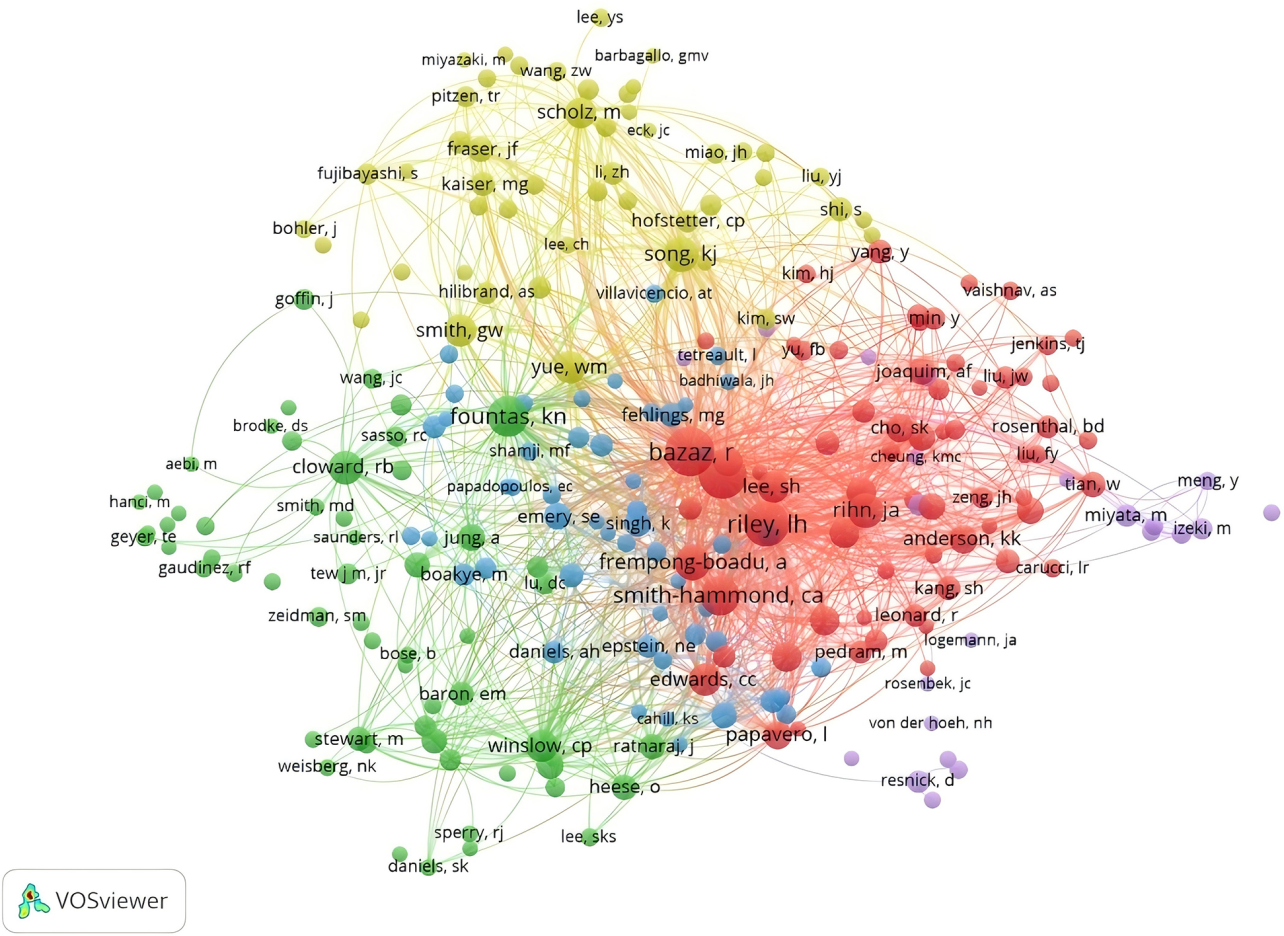
Network diagram of collaboration between authors of post-cervical spine surgery complicating dysphagia in the Web of science core collection database.

Among the top ten authors, Liu from China has the highest number of publications (*n* = 15) (Table 3), followed by Albert with 10 publications and Yang with 9. According to the network co-occurrence graphs, it can also be seen that there is a close collaboration between the authors (Figures 9, 10), among which the most cited author is BAZAZ R (*n* = 219), the only author with more than 200 citations, followed by LEE MJ (*n* = 152), RILEY LH (*n* = 135), FOUNTAS KN (*n* = 129), SMITH-HAMMOND CA (*n* = 115).

**Table 3:** Network diagram of the top 10 authors' emergence analyses of post-cervical spine surgery complicating dysphagia in the Web of science core collection database.

Rank	Authors	Counts	Rank	Cocited Authors	Counts
1	Liu, Hao	15	1	Bazazr	219
2	Albert, Todd J	10	2	Leemj	152
3	Yang, Yi	9	3	Riley LH	135
4	Singh, Kern	8	4	Fountaskn	129
5	Yuan, Wen	8	5	Smith-Hammond CA	115
6	Patel, Alpesh A	7	6	Frempong-BoadU A	90
7	Hirai, Takashi	6	7	Rihn JA	88
8	Wang, Beiyu	6	8	Song KJ	84
9	Yoshii, Toshitaka	6	9	Edwards CC	67
10	MA, Litai	6	10	Yue WM	67

### 3.6 Keyword co-occurrence, emergent analysis

**Figure 11:**
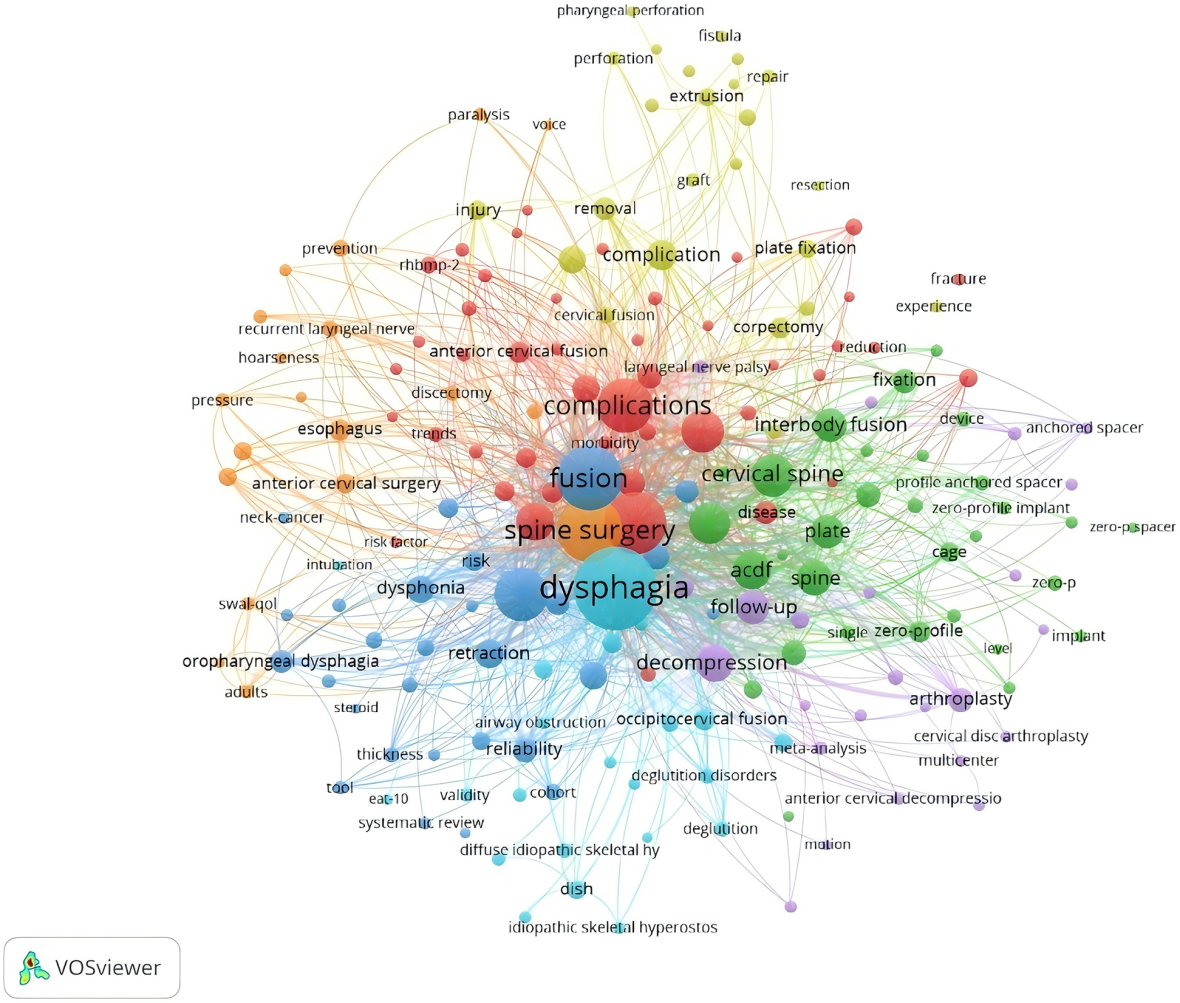
Network diagram between keywords for post-cervical spine surgery complicating dysphagia in the Web of science core collection database.

**Figure 12:**
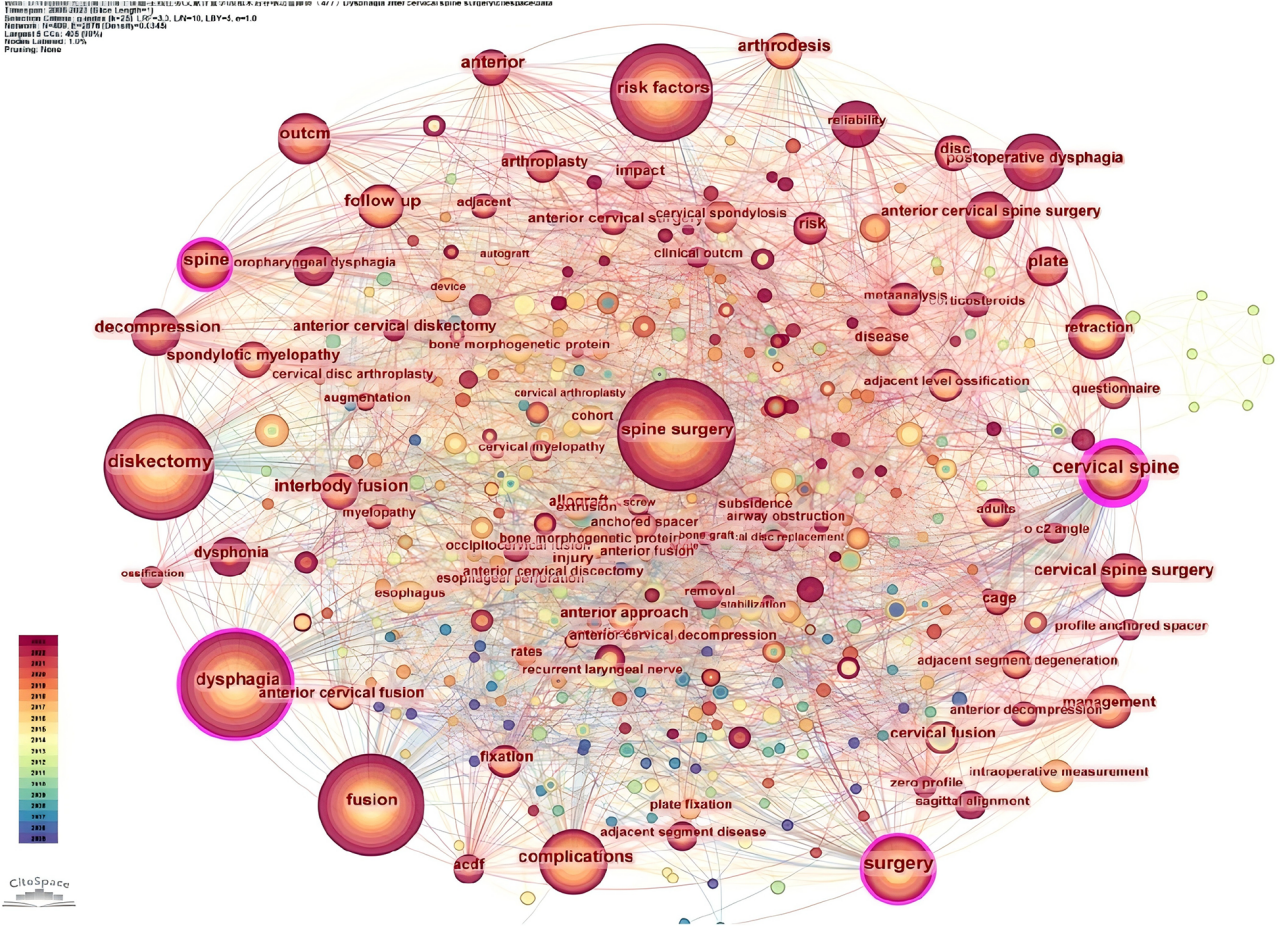
Network diagram of high-frequency keywords for post-cervical spine surgery complicating dysphagia in the Web of science core collection database.

**Figure 13:**
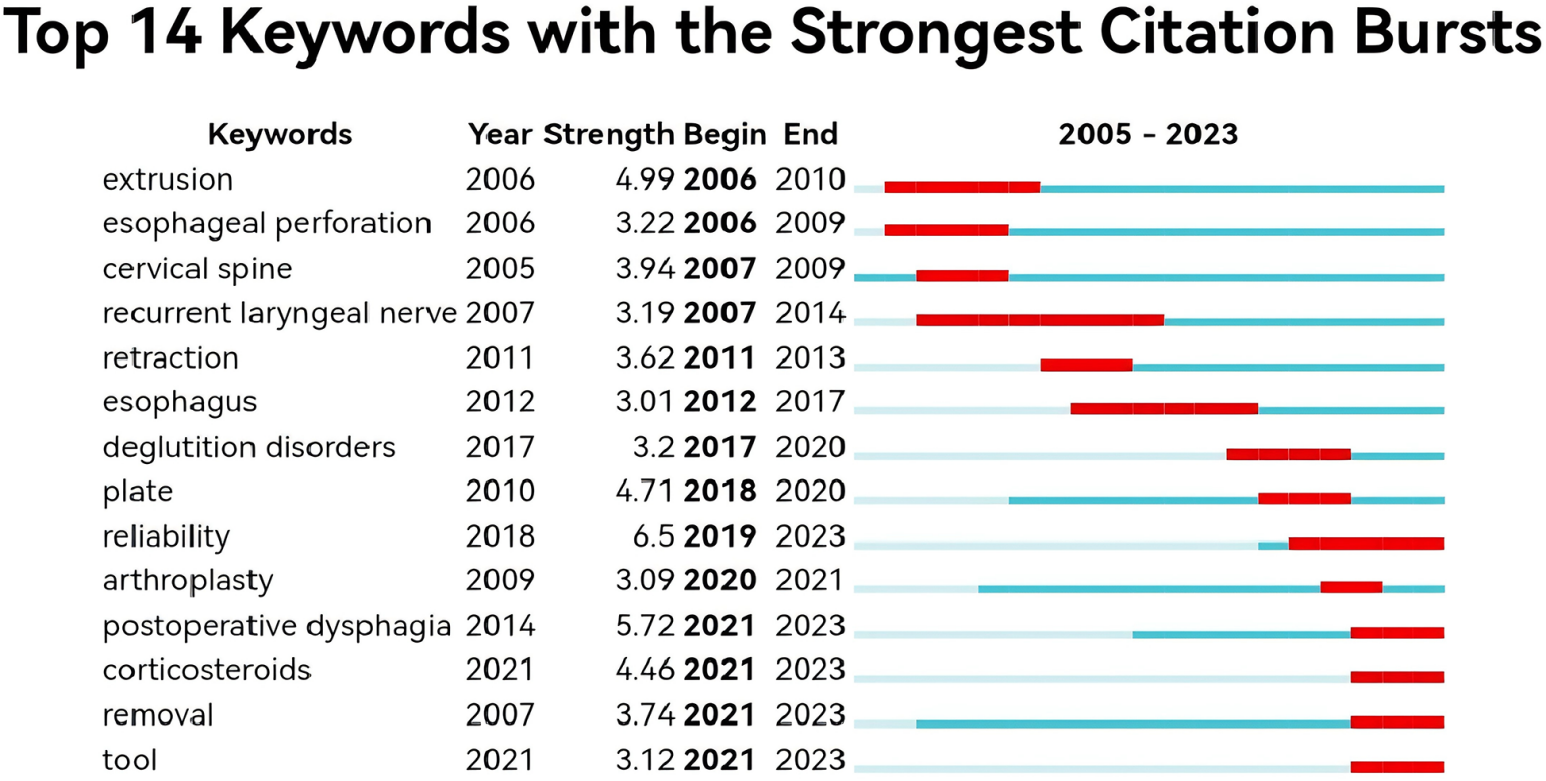
Keyword emergent network plot of postoperative dysphagia study in the Web of science core collection database.

We screened 20 high-frequency keywords (Table 4), of which the highest number was dysphagia (*n* = 303), followed by fusion (*n* = 183), spine surgery (182), diskectomy (*n* = 170), complications (*n* = 130), and the rest of them were less than 130. We used the software to analyse the network co-occurrence of the keywords, and from the figure we can see that the largest nodes are the top-ranked keywords in the table, and the lines between the different keywords are dense, which represents a high degree of correlation between each other (Figure 11). Subsequently, we used Citespace to do emergence analysis on the keywords and filtered out 14 keywords with the highest burst strength (Figures 12, 13), which were reliability (Strength = 6.5), postoperative dysphagia (Strength = 5.72), extrusion (Strength = 4.99), plate (Strength = 4.71), corticosteroids (Strength = 4.46), and cervical spine (Strength = 3.94). The largest burst of strength was reliability (Strength = 6.5), followed by postoperative dysphagia (Strength = 5.72), suggesting that it became the most popular keyword during 2019–2023.

**Table 4:** Network diagram of the top 10 high-frequency keywords for post-cervical spine surgery complicating dysphagia in the Web of science core collection database.

Rank	Keywords	Counts	Rank	Keywords	Counts
1	Dysphagia	303	11	Outcomes	60
2	Fusion	183	12	Decompression	55
3	Spine surgery	182	13	Spine	54
4	Diskectomy	170	14	Follow-up	50
5	Complications	130	15	Interbody fusion	50
6	Risk factors	125	16	Plate	48
7	Surgery	78	17	Complication	39
8	Cervical spine	76	18	Retraction	36
9	Anterior cervical discectomy and fusion	75	19	Postoperative dysphagia	34
10	Acdf	62	20	Dysphonia	34

### 3.7 Keyword time zone and cluster analysis

**Figure 14:**
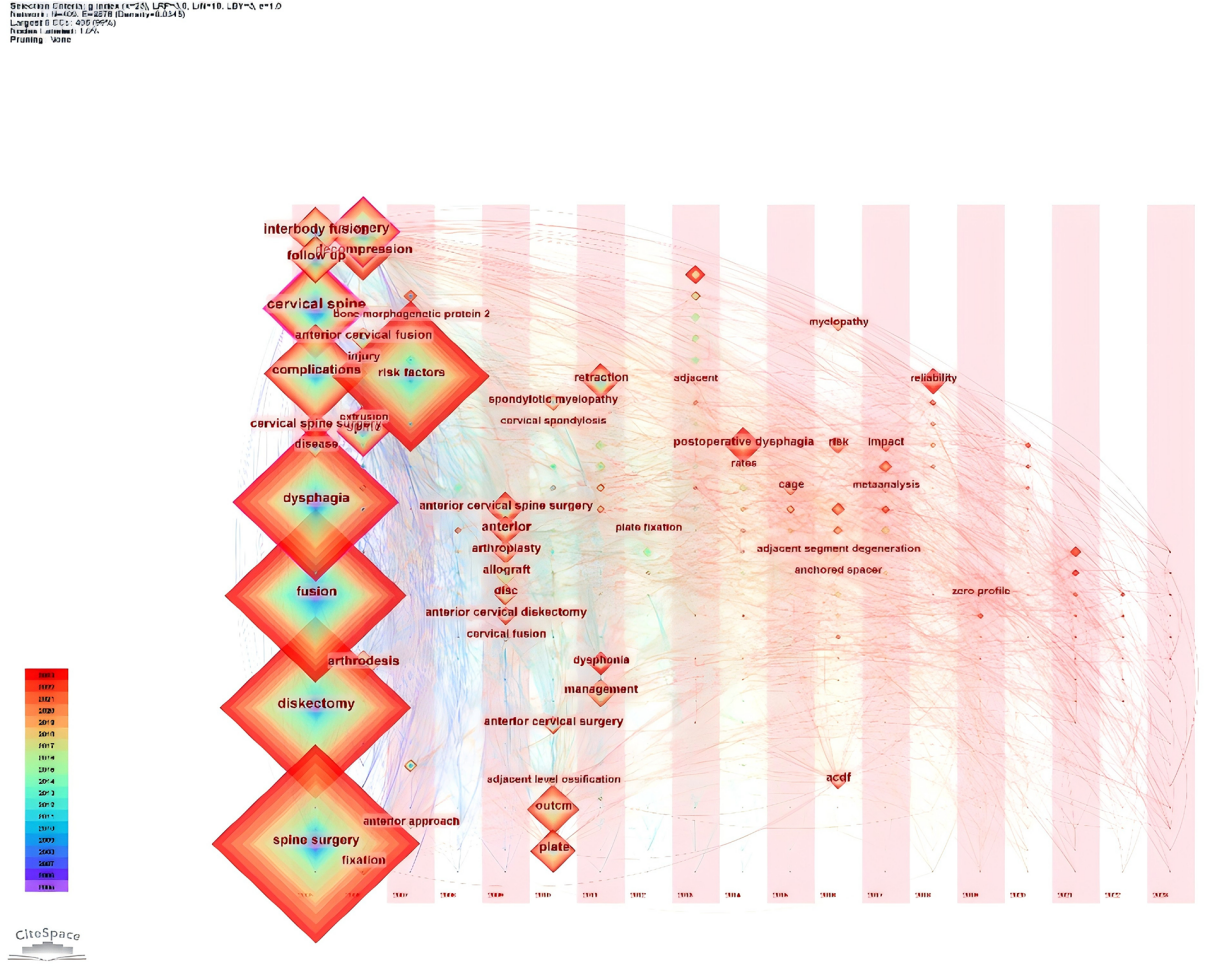
Timeline chart of keywords before dysphagia in the Web of science core collection database.

**Figure 15:**
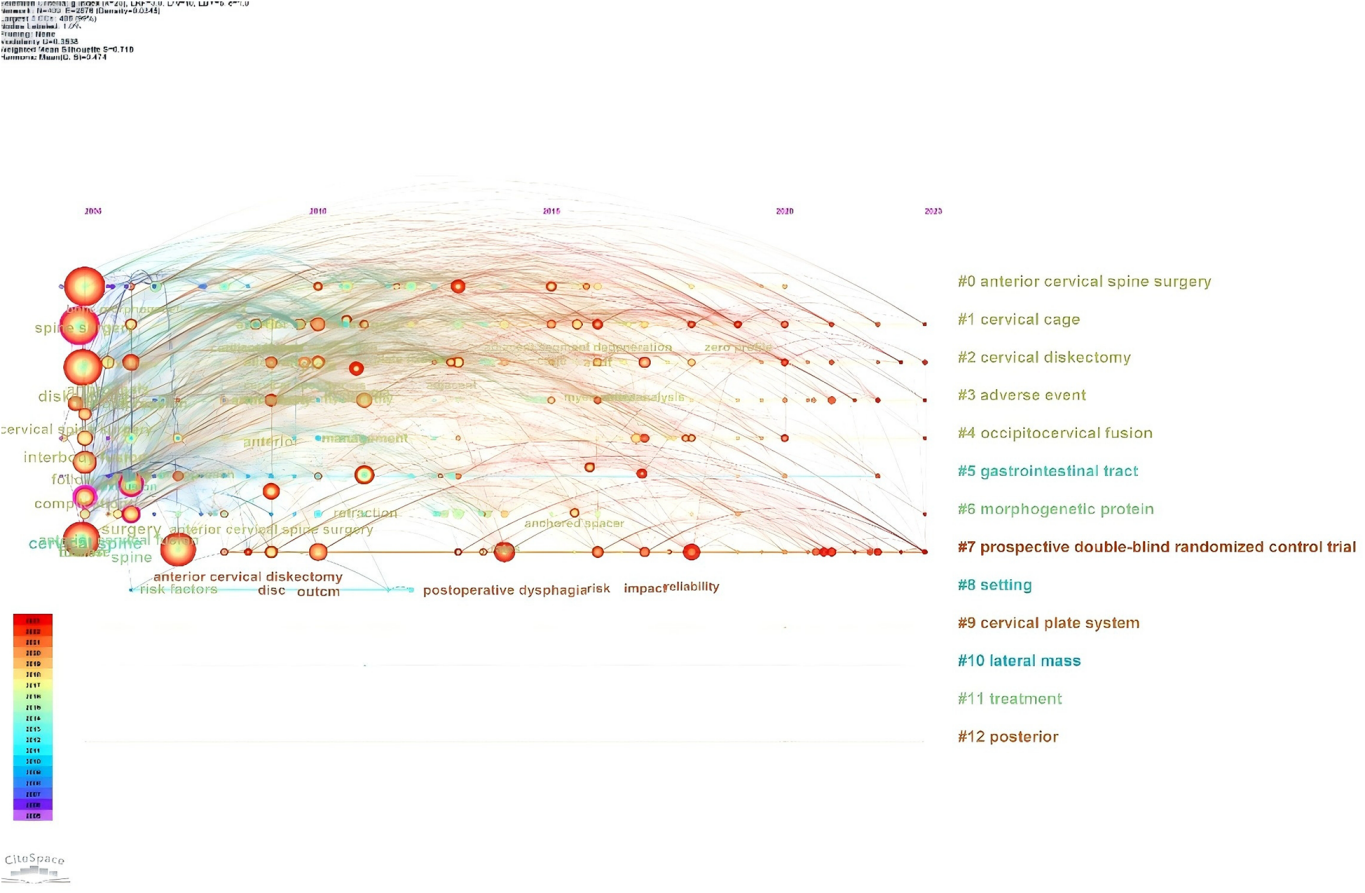
Network plot of pre-cited literature after cervical spine surgery in the Web of science core collection database.

We used Citespace to draw a keyword time zone map (Figure 14) to visualise the keywords with the corresponding years in which they appeared, from which it is obvious that the top five high-frequency keywords appeared as early as 2006, and the frequency of the appearance of this keyword has increased year by year over time, suggesting that the phenomenon of dysphagia occurring after cervical spine surgery has been a concern and a study by many scholars since as early as 2006, which indicates that many scholars began to pay attention to and conduct research on the phenomenon of dysphagia after cervical spine surgery. Subsequently, a cluster analysis of the keywords was visualised (Figure 15), where all the keywords were clustered into 13 categories, and the visualisation was plotted in relation to time, with the largest node representing the first major category (anterior cervical spine surgery), which ostensibly represents the most intensive research on the occurrence of dysphagia after cervical spine surgery in this area. The time zone and clustering analysis of keywords helped us to quickly locate the initiation time of research hotspots and research trends.

## 4 Discussion

### 4.1 Quantitative analysis of the basic information

The number of publications in the field has been at a relatively steady state over the past 20 years and reaches a peak in publication volume in 2021. The upward trend in the production of publications over the past 20 years suggests that interest in the field has increased, especially since 2020. By analysing the sources of the literature, these articles were mainly published in orthopaedic and surgical journals, with the highest number of postings in the journal Spine.The United States dominates the field of treatment of dysphagia occurring after cervical spine surgery, responding with the highest centrality as well as a distant second in the number of articles published. This is followed by China and South Korea, with only China being a developing country among the top 10 countries in terms of the number of articles published. Developed countries, such as the United States, have more favourable economic conditions than developing countries, with residents enjoying higher accessibility to insurance plans and higher economic levels (15), and therefore patients' concerns about dysphagia after cervical spine surgery are gradually increasing, which leads to more research and in-depth publications on dysphagia after cervical spine surgery in developed countries than in developing countries. Therefore, it is recommended that Chinese scholars conduct more collaborative research with other developed countries (16, 17).

Of the top 10 institutions, most are from the United States, 60 per cent are universities, and the links between institutions are more dispersed. In the author network diagram, the top two authors in terms of publications each form a research cluster centred on them, but the links between the clusters are more diffuse. There is growing evidence that more inter-institutional communication and collaboration among authors may be associated with higher research productivity and research quality (18, 19). Therefore, there is a need to expand collaborative networks between institutions and authors, especially with US universities.

### 4.2 Research hotspot analysis

Keyword clustering summarises the core and hotspots of the research areas. Dysphagia and fusion spine surgery are 3 hot areas. Currently, there are 3 main research areas in this field: interventions, influencing factors, and prognostic outcomes, but there are few studies based on how to choose treatments in different regions and ethnicities (20).

Notably, dysphagia is the most popular topic (21). Previous studies have suggested that dysphagia after anterior cervical spine surgery is due to anterior cervical soft tissue swelling caused by prolonged intraoperative stretching of the anterior cervical muscles, trachea, and oesophagus, oesophageal injury, local ischemia due to high pressure in the oesophagus, pharyngeal nerve injury from intraoperative stretching, and strain injury to the superior laryngeal or laryngeal recurrent nerves; however, the specific pathological mechanisms are not clear (22–24). Researchers should conduct high-quality randomised controlled trials of different treatment options to provide the most effective treatment regimen to improve postoperative dysphagia symptoms (25).

### 4.3 Research trend analysis

Keyword highlighting can reflect the history and frontiers of a study. From 2005–2007, the first stage, the main research content is the phenomenon of dysphagia after cervical spine surgery; 2008–2012 is the second stage of the study, the main research content is the impact of choosing different treatment modalities on patients; 2013–2015 is the third stage of the study, the main research content is the impact of dysphagia after cervical spine surgery on the quality of life of patients. 2016–2023 is the fourth stage of the study, the main research content is the treatment of cervical spine surgery. 2016–2023 is the fourth phase of the study, and the main study is about the treatment of dysphagia occurring after cervical spine surgery.

### 4.4 Literature was co-cited for analysis

Through in-depth mining and summarisation of the content of highly cited literature, the discovery of dysphagia occurring after cervical spine surgery was found, dysphagia occurring after cervical spine surgery has appeared in 2006 (26–28), and the frequency of this keyword appeared increases year by year over time, indicating that the phenomenon of dysphagia occurring after cervical spine surgery has been concerned by many scholars and researched as early as in 2006 (29). All the keywords were clustered into 13 categories, and a visualisation graph was drawn combining time, in which the largest node represented the first major category (anterior cervical spine surgery), surfacing the most intensive research on the occurrence of dysphagia after cervical spine surgery in this field. The time zone and clustering analysis of keywords helped us to quickly locate the initiation time of research hotspots and research trends (30, 31).

### 4.5 Preliminary summary

In the past 20 years, many experts have published a large number of relevant works on the occurrence of dysphagia after cervical spine surgery, but there is a lack of summary studies, which makes it difficult for researchers to find out the hotspots and research trends. Compared with what has been written by others in the past as well as in other articles, and unlike the previous method of researching for a single treatment plan, this article mainly uses bibliometric methods to summarise the relevant studies in the past 20 years and analyse the hotspots and research trends to provide a certain theoretical basis for future research. 20 years of relevant research, analysing the hotspots and research trends of dysphagia occurring after cervical spine surgery, and providing a certain theoretical basis for future research. However, the article also has certain defects, and there are personal subjective judgements in the literature collection and the formulation of the search formula. It is suggested that the correlation between curvature and prognosis should be strengthened in the future, which will have positive results in predicting the outcome of treatment. In addition, research on the etiology of dysphagia after cervical spine surgery should be strengthened in order to help target treatment in the future.

### 4.6 Article limitations

Firstly, the article only searched publications from Web of Science core set databases, and for other large databases such as China Knowledge, PubMed or Embase from which literature related to dysphagia disease occurring after cervical spine surgery was not included. Due to the different attributes of different databases (32), it may not be appropriate to combine papers from multiple databases. In addition, the Web of Science Core Collection database is the most representative and cutting-edge authoritative database of all databases, and it contains the best-known high-impact academic journals in the world. Secondly, limited by the CiteSpace software, the article only includes articles published in English in the past 20 years, which is not comprehensive enough for the collection of literature. Due to the time of completion of the article, literature published since the completion of the literature search was not included.

### 4.7 Conclusion

The article provides a visualisation of the research and analysis of dysphagia occurring after cervical spine surgery. Research related to dysphagia occurring after cervical spine surgery has remained relatively stable over the period 2017–2023, and by analysing and discussing the collaborative nature between countries, authors, and institutions, it was found that enhanced collaboration between countries, institutions, and authors can have a positive effect on the output of high-quality research and promote in-depth research on dysphagia occurring after cervical spine surgery, especially Developing countries should actively collaborate more with developed countries. Through in-depth discussion and analysis of keyword clustering, it is found that the current research hotspots are dysphagia, fusion and spinal surgery, and strengthening the research on this hotspot will help to have a more accurate prediction of dysphagia after cervical spine surgery. Through the analysis and mining of keyword emergence, it was found that the relationship between curvature and prognosis and the etiology of dysphagia after cervical spine surgery are considered to be new research trends in the future, and that strengthening the research on the relationship between curvature and prognosis and the etiology of dysphagia after cervical spine surgery can provide new therapeutic ideas and personalised treatment plans for dysphagia after cervical spine surgery, which can positively impact on the treatment of dysphagia after cervical spine surgery. The article facilitates the transition of the research results to clinical practice. The article facilitates the transition of research results to clinical practice and provides a reference for scholars to conduct further research.

## Data Availability

The original contributions presented in the study are included in the article/Supplementary Material, further inquiries can be directed to the corresponding author.
